# Prediction of the potential global distribution for *Biomphalaria straminea*, an intermediate host for *Schistosoma mansoni*

**DOI:** 10.1371/journal.pntd.0006548

**Published:** 2018-05-29

**Authors:** Ya Yang, Wanting Cheng, Xiaoying Wu, Shaoyu Huang, Zhuohui Deng, Xin Zeng, Dongjuan Yuan, Yu Yang, Zhongdao Wu, Yue Chen, Yibiao Zhou, Qingwu Jiang

**Affiliations:** 1 Key Laboratory of Public Health Safety, Ministry of Education, Tropical Disease Research Center, Department of Epidemiology, School of Public Health, Fudan University, Shanghai, China; 2 Institute of Parasitic Diseases, Guangdong Provincial Center for Disease Control and Prevention, Guangdong, China; 3 Department of Parasitology, Zhongshan School of Medicine, Sun Yat-sen University, Guangdong, China; 4 School of Epidemiology and Public Health, Faculty of Medicine, University of Ottawa, Ottawa, Canada; Centers for Disease Control and Prevention, UNITED STATES

## Abstract

**Background:**

Schistosomiasis is a snail-borne parasitic disease and is endemic in many tropical and subtropical countries. *Biomphalaria straminea*, an intermediate host for *Schistosoma mansoni*, is native to the southeastern part of South America and has established in other regions of South America, Central America and southern China during the last decades. *S*. *mansoni* is endemic in Africa, the Middle East, South America and the Caribbean. Knowledge of the potential global distribution of this snail is essential for risk assessment, monitoring, disease prevention and control.

**Methods and findings:**

A comprehensive database of cross-continental occurrence for *B*. *straminea* was compiled to construct ecological models. We used several approaches to investigate the distribution of *B*. *straminea*, including direct comparison of climatic conditions, principal component analysis and niche overlap analyses to detect niche shifts. We also investigated the impacts of bioclimatic and human factors, and then used the bioclimatic and footprint layers to predict the potential distribution of *B*. *straminea* at global scale. We detected niche shifts accompanying the invasions of *B*. *straminea* in the Americas and China. The introduced populations had enlarged its habitats to subtropical regions where annual mean temperature is relatively low. Annual mean temperature, isothermality and temperature seasonality were identified as most important climatic features for the occurrence of *B*. *straminea*. Additionally, human factors improved the model prediction (*P*<0.001). Our model showed that under current climate conditions the snail should mostly be confined to the tropic and subtropic regions, including South America, Central America, Sub-Saharan Africa and Southeast Asia.

**Conclusions:**

Our results confirmed that niche shifts took place in the invasions of B. straminea, in which bioclimatic and human factors played an important role. Our model predicted the global distribution of *B*. *straminea* based on habitat suitability, which would help for prioritizing monitoring and management efforts for *B*. *straminea* control in the context of ongoing climate change and human disturbances.

## Author summary

*Biomphalaria straminea* is an intermediate host of *Schistosoma mansoni*. This snail has not only established in peripheral countries but also survived in different water habitats in Hong Kong and adjacent cities of mainland China. Ecological niche models were used to predict the potential global distribution of *B*. *straminea*. Our results showed that there were niche shifts in the process of invasion for *B*. *straminea* in the Americas and China. This snail has expanded its habitats to subtropical regions with lower annual mean temperature. Annual mean temperature, isothermality, temperature seasonality and human influence were identified as most important climatic features for the occurrence of *B*. *straminea*. Under current climate conditions the distribution of this snail should be mostly confined to the tropic and subtropic regions, including South America, Central America, Sub-Saharan Africa and Southeast Asia. With the rapid globalization and the continued burden of imported cases of *S*. *mansoni* to non-endemic countries, predicting the potential distribution of the intermediate host and its drivers is increasing in importance for designing control strategies and optimizing use of limited public health resources. Prioritizing surveillance and control efforts to high-traffic regions with high habitat suitability may be the most effective approach.

## Introduction

Invasive species can often pose threats to the ecosystem functioning and biodiversity at the global scale, especially when they spread diseases[1]. There are a growing number of studies conducted for risk assessment, monitoring and management of invasive species and reduction of negative impacts. For many invasive species, however, once they are established over large areas, their eradication or removal can be an impossible task[2]. The prevention of introduction and establishment is therefore thought to be the most cost-effective way of mitigating future negative consequences[3, 4]. An important approach to prevention is predicting species with invasive tendency and areas vulnerable to their invasion, which then can guide early detection and rapid response efforts against invasive species[4–7].

*B*. *straminea* (Dunker, 1848) is a freshwater snail in the family *Planorbidae*, originated from the southeastern part of South America[8]. It is a highly invasive and competitive species given its capacity to survive during the periods of drought and its great fertility[8–10]. During the last decades, free-ranging populations of *B*. *straminea* have been reported in peripheral countries including Paraguay[11], Argentina[12], Uruguay in 1987[13], Colombia in 1966[14] and Costa Rica in 1976[15]. In the Caribbean area, its introduction has been documented in several islands of the Lesser Antilles, namely Martinique around 1950[16], Grenada in 1970[17], Guadeloupe in 1985[18] and St Lucia in 1992[19]. Over the same period, the snail has invaded several new states in Brazil and replaced other Biomphalaria species following its introduction[20]. In addition, *B*. *straminea* is noted for its long-distance dispersion and establishment in Hong Kong in 1974, on the Pearl River Delta of China[21]. The snail subsequently dispersed to different water habitats in adjacent cities in Guangdong Province of southern China, including Shenzhen, Dongguan and Huizhou[22].

*B*. *straminea* is an intermediate host of *Schistosoma mansoni*[9, 23, 24], and is one of the three species found to be naturally infected with *S*. *mansoni* in Brazil[8]. *S*. *mansoni* is a snail-bone parasitic disease, prevalent predominantly in Africa, the Middle East, the Caribbean, Brazil, Venezuela and Suriname[25]. China started several aid programs in African countries in the 1970s. Since then, imported cases of *S*. *mansoni* from Africa have been increasing, which has captured much attention from public health officials[25]. The existence of imported patients and its intermediate host is the prerequisite of transmission of *S*. *mansoni* in China. Moreover, the increasing amount of logistics and human flows induced by the Belt and Road Initiative would put China at a greater risk of the disease[26]. Furthermore, global warming is thought to change the current habitats of *B*. *straminea*, thereby affecting the original landscape of schistosomiasis[25]. *B*. *straminea* was also found to precede the common snails as a carrier of *Angiostrongylus cantonensis*, an important neurotropic pathogen of human angiostrongyliasis, under laboratory conditions.[27] The study of potential distribution and suitable habitats of this snail are therefore of particular importance for global health.

Ecological niche modeling is increasingly used to predict the distributions of species and vector-borne diseases[28–32]. This modeling method can not only predict distributional ranges, but also identify what particular combination of environmental variables shapes a species’ distribution[4, 29]. Previous studies have utilized ecological models to predict the spatial distribution of *B*. *straminea* at state and national scales in Brazil[9, 24]. There is only one study that built the prediction map in China based on the occurrence data in Shenzhen city[33]. However, species distribution models, which do not incorporate data from both the native and introduced ranges, likely misrepresent the potential distribution of invasive species, especially under projected climate change scenarios[34]. The aim of this study was threefold. First, we compared the niches of the native and introduced populations to assess whether niche shifts occurred in the invasion of *B*. *straminea*. Second, we investigated the impacts of bioclimatic and human factors in the process of invasion. Third, we used pooled data from both the native and introduced ranges to predict the potential distribution of *B*. *straminea* at a global scale.

## Methods

### Occurrence records

We obtained occurrence records from a comprehensive literature review (S1 Table), the Global Biodiversity Information Facility database (GBIF, http://www.gbif.org/, last accessed December 2016), and results from our own malacological surveys. All available location information was extracted for each occurrence. We georeferenced records from literature and GBIF that had only the administrative region using Google Maps (http://www.google.cn/maps), Google Earth (https://www.google.com/earth/), or simple Google searches. We overlaid the geolocated occurrence points with a raster layer that distinguished land from water. Any records that were positioned outside the land area were subsequently removed from the database.

Malacological surveys were conducted during the period from 2012 to 2016 in the river systems of cities (Guangzhou, Shenzhen, Dongguan and Huizhou) adjoining Hong Kong, in which the first introduction of *B*. *straminea* in China was reported. Sampling was carried out by two trained field investigators using standard snail scoops. At each site, the investigators collected any *Biomphalaria* snails found in a radius of approximately 2 m over a permitted search time of 30min. The coordinates of each sampling sites were recorded with the help of a handheld geographical positioning systems (GPS) device (Trimble Navigation Co., Ltd.). Collected snails were appropriately labeled, transported to the laboratory and identified using the morphological approach. Key characters were shape of the shells and number of the prostate diverticula as previously described[8].

**Fig 1:**
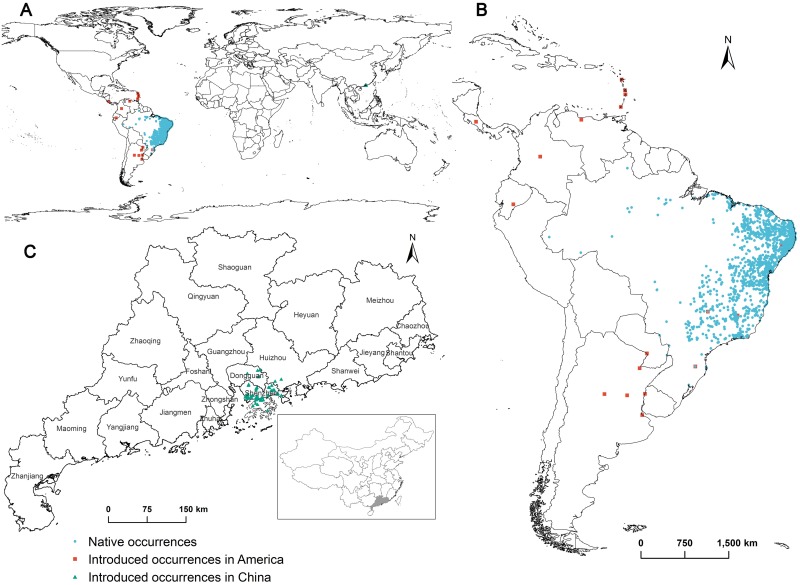
Geographical distribution of *B*. *straminea* occurrence records. In panels A, B and C, blue dots indicate occurrence records from the native range, red squares are records from introduced ranges from the America and green triangles from China.

To minimize the effect of sampling bias, we retained only one occurrence point per 2.5 arc-min resolution grid (a 5×5 km area)[30, 35, 36]. The final data set included 1312 occurrence points (1262 from the native range, 19 from introduced ranges in the Americas and 31 from China) (Fig 1).

### Environmental variables

We obtained 19 bioclimatic variables with a spatial resolution of 2.5 arc-min from WorldClim version 1.4[37]. The bioclimatic variables represent annual trends, seasonality and extreme environmental factors. They have been widely applied to model the ecological niche and potential distribution of species[30, 35, 38, 39].

We also included the global human footprint layer version 2 (Wildlife Conservation Society (WCS), & Center for International Earth Science Information Network (CIESIN)) in our model to evaluate the correlation between anthropogenic influences and the distribution of the introduced occurrences. The human footprint layer measures the human influence on global surface, combining data sets representing human population density, land transformation, human access, and presence of infrastructures[38, 40]. We used the same resolution for this layer as for the bioclimatic variables.

To reduce the effects of overparameterization and multicollinearity of predictors, we calculated the Pearson’s correlation coefficient for each pairwise comparison for all 19 bioclimatic variables and the human footprint, and excluded variables with a high intercorrelation (r > 0.90) (S1 Fig). The final environmental data set included 13 variables (S2 Table).

### Comparisons of native and invaded ecological niches

We applied three different methods to test the differences in the variables of bioclimatic environments at the occurrences between the native and introduced regions. First, we extracted the values of predictors for each occurrence and used the Kruskal-Wallis test to compare the pairwise differences in the distribution of each variable between the three distributional records. *P*-values were Bonferroni-corrected to avoid false significant differences. Second, we employed principal component analysis (PCA) to compare the position of occurrences from the native and invaded ranges in the bioclimatic space[30, 41]. Third, we calculated the Schoener’s index for niche overlap (D) for each pair of occurrences of *B*. *straminea* snails. D ranges from 0 (no overlap) to 1 (identical). We then used niche equivalence and similarity tests that rely on the metric D to detect niche shifts[42, 43]. We used a buffer of 500 km around each known occurrence, which would provide better model predictions[44]. Computations of D, niche similarity and equivalence were performed using the ENMTools package in R.

### Ecological modeling and evaluation

Modeling was conducted using Maxent (3.3.3k, http://biodiversityinformatics.amnh.org/open_source/maxent/), which is a widely used machine learning algorithm that estimates the species’ probability distribution of maximum entropy, constrained by incomplete information about the species’ distribution and the environmental factors[29]. Maxent generally has a better performance for presence-only data sets and is particularly robust at small sample sizes in comparison with other species distribution models[28, 45, 46].

To test the contribution of human impacts on the distribution of *B*. *straminea*, we built two models for all occurrences using twelve bioclimatic layers with and without the human footprint layer. Default settings were used when not otherwise stated. Each model was replicated ten times so that results were summarized as an average of the ten models. Occurrence data were randomly split into a training subset (75%) and a test one (25%). Subsequently, model averages were projected on a global scale under current climatic conditions. Prediction maps were generated using the tmap package in R.

We used two metrics to evaluate the model prediction performances: the area under the curve of the receiver operating characteristic (AUC) and the sample size corrected Akaike’s Information Criterion (AICc). The AUC uses presence and absence records to assess model predictive performance across a range of thresholds. The AUC ranges from 0 to 1, where a score of 0.7–0.8 is thought to be an acceptable prediction, 0.8–0.9 is good and >0.9 is excellent[28]. The AICc can outperform AUC as a model selection criteria, particularly when sample sizes are small[47]. A lower AICc value indicates better model fits. We then compared the means of the AUC and AICc values using t-test to assess whether the human footprint could increase the predictive ability significantly. We also evaluated the relative contribution of each variable to the model.

## Results

### Comparisons of bioclimatic conditions between the native and introduced occurrences

**Fig 2:**
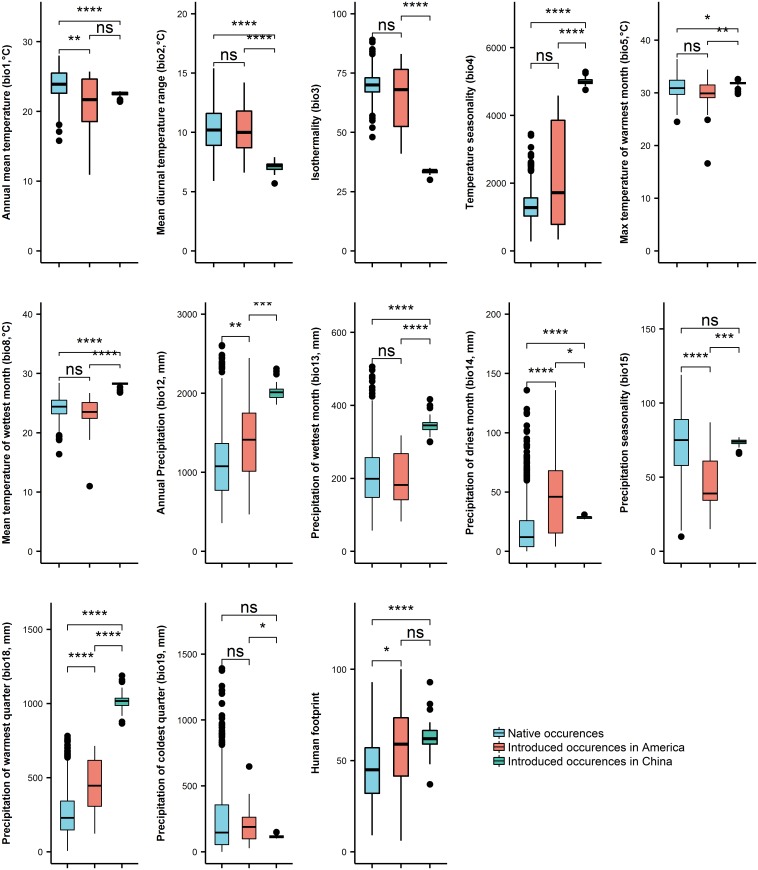
Comparison of bioclimatic variables between native ranges of native occurrences (blue), introduced occurrences in the Americas (red) and introduced occurrences in China (green). Asterisks (*) indicate significant pairwise Kruskal-Wallis test differences between the three ranges (ns: not significant, *P < 0.05, **P < 0.01, ***P < 0.001, ****P < 0.0001).

Fig 2 summarizes the pairwise comparisons of bioclimatic conditions for *B*. *straminea*. For the native and introduced occurrences in the Americas, only annual mean temperature (bio1) was lower in the introduced range than in the native range whereas differences of the other temperature-related variables were not significant. The introduced range had higher annual precipitation (bio12), precipitation of driest period (bio14), precipitation of warmest quarter (bio18) and human influence than the native range but lower precipitation seasonality (bio15).

Annual mean temperature (bio1) was lower in the introduced range in China than in the native range with lower isothermality (bio3) and mean diurnal temperature range (bio2). The introduced range in China had higher temperature seasonality (bio4) and higher temperatures in the warmest period (bio5) and in the wettest quarter (bio8) than the native range. Anthropogenic impacts on the environment were higher in the introduced region in China with higher annual precipitation (bio12) and precipitations in all seasons except the coldest quarter (bio13, bio14, bio18).

Annual mean temperature (bio1) and isothermality were lower in the introduced range in China than in the introduced range in the Americas. The introduced range in China had higher temperature seasonality (bio4) and higher temperatures in the warmest period (bio5) and in the wettest quarter (bio8) than the introduced range in the Americas. The introduced ranges in China and the Americas also differed in all the precipitation requirements (bio12-15, bio18 and bio19), but seemed similar in human impacts on the environment.

### Principal component analysis

**Fig 3:**
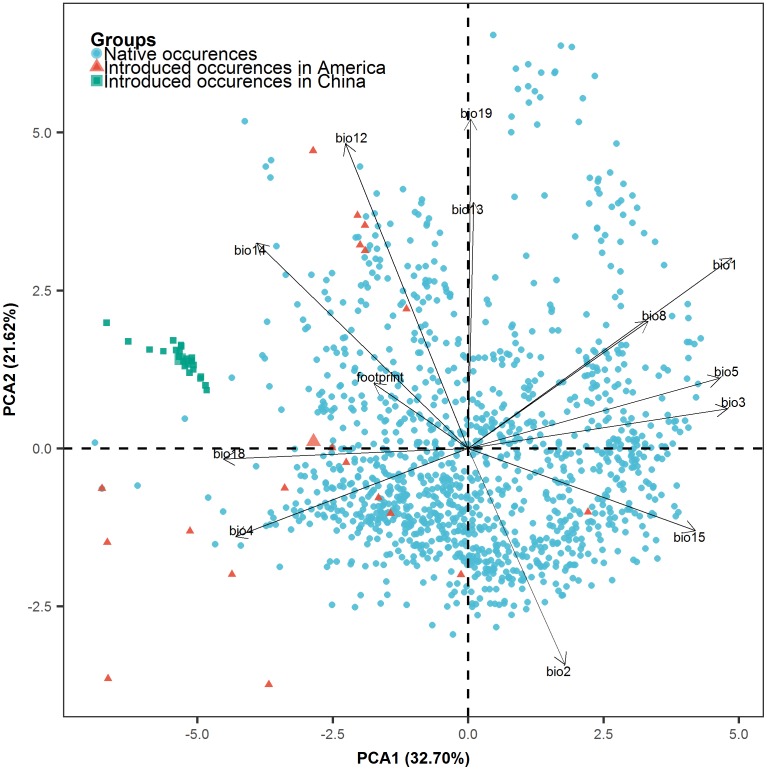
Biplot showing the bioclimatic spaces for the three groups of occurrence records of *B*. *straminea*. The line length indicates the importance of each variable on the two axes of the principal component analysis.

Principal component analysis of the predictors revealed two significant axes of climatic variation, which explained 54.32% of the total variance. The first principal component (PCA1) was closely related to both temperature and precipitation while the second one was mostly associated with precipitation (Fig 3, Table 1). The environmental space occupied by the introduced population of *B*. *straminea* in the Americas (red triangles) largely overlapped with that of the native occurrences (blue dots) (Fig 3). The niche shift of the introduced American population (red triangles) occurred principally along axis 1, indicating different component weights of each variable as the underlying gradient of niche differentiation. The pattern of the introduced population in China was more obvious, as they (green squares) clustered together and appeared isolated from both the native and nonnative American occurrences (Fig 3). Human impacts appeared to be an important factor for the occurrences in China, as the China cluster shifted roughly along the direction of human footprint variable (Fig 3).

**Table 1:** Principal component weights of each bioclimatic variable assigned by the principal component analysis.

Variables	Component 1	Component 2	Component 3	Component 4
bio1	0.79	0.49	0.05	-0.33
bio2	0.29	-0.55	0.45	0.32
bio3	0.77	0.10	-0.38	0.36
bio4	-0.69	-0.23	0.50	-0.32
bio5	0.75	0.18	0.40	-0.36
bio12	-0.37	0.78	0.34	0.31
bio13	0.02	0.63	0.57	0.49
bio14	-0.63	0.53	-0.32	-0.22
bio15	0.68	-0.21	0.48	0.31
bio18	-0.73	-0.03	0.57	0.08
bio19	0.01	0.84	-0.31	0.13
footprint	-0.28	0.17	0.34	-0.08
Eigenvalue	4.25	2.81	2.33	1.34
Cumulative percentage of variance	32.7	54.32	72.21	82.5

### Niche similarity tests

All occurrence pairs showed very limited levels of niche overlap (Schoener’s D between 0.03–0.17), with the highest overlap found between the native range and the introduced range in the Americas (Table 2). The null hypotheses of niche equivalency and similarity were rejected for all occurrence pairs (P<0.05).

**Table 2:** Analyses of niche overlap, equivalency and similarity of climatic niches based on the distributions of *B*. *straminea*.

Occurrence Pairs	Schoener’s D	P value (Equivalency test)	P value (Similarity test)
Native-Nonnative America	0.17	0.01	0.02
Native-China	0.03	0.01	0.01
Nonnative America-China	0.04	0.01	0.01
America combined-China	0.03	0.01	0.01

### Ecological modeling and variable contributions

**Fig 4:**
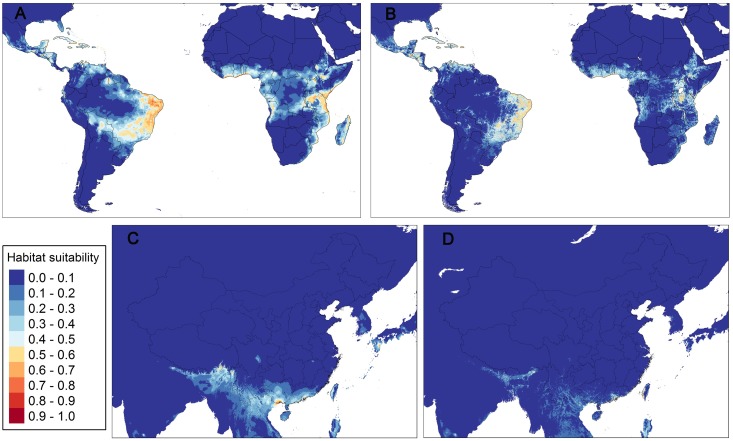
Global potential distributions of *B*. *straminea* in America, Africa (A&B) and China (C&D). Panels A and C were outputs without the human footprint layer while B and D were built with this layer. The scores indicate the environmental suitability.

The ecological models performed reasonably well, with all AUC values >0.9. The predictive capability was significantly higher for models with the human footprint layer (full model) than those without (P < 0.001). The AICc of the full model was significantly lower than the model with bioclimatic variables only (P < 0.001), indicating that the human footprint layer improved the model performance significantly (Table 3). The prediction map of the full model also showed less medium suitable areas (light blue) than the model without the human footprint layer (Fig 4). In China, the two models differed most in the southern regions, including Guangxi and Guangdong Provinces. In addition to southern China, the most suitable regions for the establishment of *B*. *straminea* were identified in the eastern regions of South America, Central America, western and eastern regions of Africa and Southeast Asia (Fig 4).

**Table 3:** Model performances and results of t-tests. Data are shown as means (SD) of ten runs.

Metrics	Without footprint layer	With footprint layer	P value
AUC	0.932 (0.004)	0.941 (0.004)	<0.001
AICc	34981.960 (9.123)	32505.900 (0.206)	<0.001

The inclusion of the human footprint layer influenced the relative contributions and permutation importance of the bioclimatic variables (Table 4). The human footprint, isothermality (bio3) and temperature seasonality (bio4) had higher relative contribution in predicting the distribution of *B*. *straminea*. Annual mean temperature (bio1), isothermality (bio3) and the human footprint were the most important judging by the metric of permutation importance.

**Table 4:** Relative contributions and permutation importance of bioclimatic variables and human footprint to the current predicted distribution of *B*. *straminea*. Data are shown as means (SD) of ten runs (%).

Variables	Relative contribution		Permutation importance	
	Without footprint layer	With footprint layer	Without footprint layer	With footprint layer
bio1	1.905 (0.484)	1.717 (0.287)	22.194 (2.962)	21.287 (2.590)
bio2	6.396 (0.434)	0.849 (0.229)	3.859 (0.362)	1.169 (0.221)
bio3	43.312 (1.334)	31.944 (2.052)	22.320 (2.008)	26.625 (3.474)
bio4	30.272 (1.367)	17.971 (1.635)	22.168 (1.451)	4.457 (0.696)
bio5	3.253 (0.255)	2.233 (0.413)	8.718 (0.483)	5.585 (0.709)
bio8	0.691 (0.114)	0.975 (0.738)	3.258 (0.770)	2.929 (1.205)
bio12	9.182 (0.532)	1.790 (0.618)	8.330 (0.390)	5.190 (0.430)
bio13	0.030 (0.006)	0.164 (0.211)	0.709 (0.118)	0.127 (0.041)
bio14	2.173 (0.242)	1.209 (0.177)	1.492 (0.158)	0.554 (0.126)
bio15	0.522 (0.110)	0.164 (0.194)	1.812 (0.197)	0.464 (0.070)
bio18	1.935 (0.145)	0.938 (0.110)	4.284 (0.157)	3.815 (0.387)
bio19	0.327 (0.130)	0.317 (0.223)	0.855 (0.151)	0.452 (0.120)
footprint	-	39.729 (1.217)	-	27.345 (1.153)

## Discussion

Considering the distribution of *B*. *straminea* in its native range and invaded peripheral regions in the Americas, it has a broad geographical and climatic range (Figs 1, 2 and 3). Like other invasive species, *B*. *straminea* snails have developed a variety of survival strategies to adapt to variable environments[9]. Our study has demonstrated that the *B*. *straminea* occupies significantly different ecological niches in the native region and invaded regions in the Americas and China, providing empirical evidence of the ability of this type of invasive snails to repeatedly acclimate to new environmental conditions. Such capability may be a fundamental basis for a successful invasion of exotic species[30]. The shifts were significant for the two groups of invaded occurrences in the Americas and China. Although for these two ranges, there were lack of evidence for niche identity and similarity, the climatic niche shifted towards a similar direction (Fig 2).

There are potential mechanisms for the observed niche shifts in our study, which include: 1) snail breeding conditions in the invaded range that are not accessible in the native range due to geographical dispersal limitations; 2) a biotic release from native predators, competitors and pathogens or diseases in the novel habitats; and 3) a rapid evolution accompanied the invasion, which may allow them to colonize new environments[48–50]. Unfortunately, disentangling the relative roles of these mechanisms is not possible based on our data. Further field and experimental studies are required to clarify the mechanisms behind the climatic niche shifts for *B*. *straminea* invasions.

Our results indicated that the annual mean temperature, isothermality and temperature seasonality were the most important climatic features for the occurrence of *B*. *straminea*. The snail can be found in a wide variety of shallow aquatic habitats with little water velocity, such as small pools, lakes, streams, irrigation channels and flooded areas[8]. Fluctuations of water temperature may have a direct effect on the growth of juvenile snails as well as the reproduction and survival of adults[8, 51]. They keep active at a temperature of 18–32 °C while the optimum temperature for their development is 20–26 °C[51]. In some permanent habitats with compatible temperature, *B*. *straminea* reproduces throughout the year; in others with short window of suitable temperature, only a single generation is produced each year[51]. In addition, temperature is associated with the production and release of schistosome cercariae[8]. The climate of the native and the invaded Caribbean area and Colombia is tropical but the other invaded regions, including Paraguay, Argentina, Uruguay, Hong Kong and coastal mainland China, have a subtropical climate characterized by humid, hot summers and relatively dry and mild winters. Evidently, *B*. *straminea* has expanded its domain to regions with lower annual temperature partially facilitated by its ability to survive adverse environmental conditions, e.g. lower or higher temperature and desiccation, in the mud[51]. Annual precipitation also played a role in the occurring of *B*. *straminea*, which was consistent with a recent survey that found a positive correlation between precipitation and *B*. *straminea* abundance[52].

Inclusion of the human footprint layer enhanced the accuracy of the ecological niche model, indicating that human activities can contribute to the spreading of introduced *B*. *straminea*. Our finding supported the association between human-intervened environmental changes and distribution of schistosomiasis and its intermediate hosts[53, 54]. The development and management of water resources is thought to be an important risk factor for schistosomiasis transmission[55]. Human activities can affect the biological invasions by changing the spreading routes of the species and creating suitable microclimates in regions with suboptimal climates[56]. Non-native species tend to colonize man-made suitable habitats first and move on to natural environments[57]. The first introduction of *B*. *straminea* into Hong Kong was thought to be associated with imported tropical aquarium plants or fish from South America[22, 58], which was reconfirmed by a recent phylogenetic analysis[59]. Subsequent colonization occurred in large outdoor concrete breeding ponds for tropical aquarium fish near the border with mainland China[60]. This snail was first found in several ponds, ditches and rivers in Shenzhen of China, and spread further in the Shenzhen River system[58]. Shenzhen was one of the fastest-growing cities in the world and has undergone magnificent landscape reconstructions, which may lead to dispersion of the snails to surrounding cities[61]. A survey conducted in 2012–2013 noted that this snail had established in several waterways in Dongguan and Huizhou[61]. Therefore, environmental assessment is advised for infrastructure projects, especially water conservancy schemes, that might transport *B*. *straminea* to novel habitats.

According to our model, *B*. *straminea* showed a variable range of suitable areas in the tropical and subtropical regions including continental and coastal areas and islands. The most suitable regions for invasion covered Central America, Sub-Saharan Africa and Southeast Asia (Fig 4). *B*. *straminea* is present in several countries in Central America, but its role as the intermediate host of *S*. *mansoni* has never been confirmed in these countries[11]. Africa is home to other intermediate hosts of *S*. *mansoni*, including *B*. *pfeifferi*, *B*. *alexandrina* and *B*. *sudanica*[62]. Although this continent has no evidence so far for the presence of *B*. *straminea* snails, our predicted suitable habitats largely overlap with the distribution of *S*. *mansoni* in Africa[63]. Once the *B*. *straminea* was introduced and successfully established in these regions, it might complicate the life cycle of *S*. *mansoni* and increase the burden of schistosomiasis control for African countries. Therefore, in African regions efforts should be mainly directed to monitoring water bodies for early signs of an invasion by global trade and transport.

Identified suitable areas in China were constrained to tropical and subtropical regions. However, the low suitability of some areas does not mean that *B*. *straminea* can be introduced without any risk of invasion since these snails could breed in suitable artificial microhabitats. Moreover, the ongoing global climate change, in particular global warming, can modify the suitability and cause an expansion of suitable areas towards higher latitude[25]. The outbreak of urogenital schistosomiasis in Corsica, France, sounded the alarm and suggested that the transmission cycle of schistosomiasis can be completed upon the encounter between the intermediate host snails and imported individuals infected the parasite[64]. Since the 1970s, imported cases of *S*. *mansoni* or *S*. *haematobium* has been repeatedly reported among migrant workers, who are at high risk of African schistosome infections because of frequent contact with infested water. Few of the individuals infected with *S*. *mansoni*, usually characterized by only mild or no symptoms, seek medical help and they may be misdiagnosed for the reason that Chinese clinicians lack knowledge of the diagnosis of this disease. The priorities of China’s health authorities are to monitor and block further spread of *B*. *straminea* as well as to develop strategies to reduce the imported cases of *S*. *mansoni* from endemic areas.

In conclusion, our study showed that the environmental spaces of the introduced *B*. *straminea* populations in the Americas and China shifted compared to that of their native counterparts. This snail has acclimated to parts of the subtropics with lower annual mean temperature. Incorporation of anthropogenic factors improved niche model prediction in areas of high human disturbance. Our final model predicted large suitable areas in the tropics and subtropics, indicating that *B*. *straminea* snail has a significant potential to spread further as nonnative species. Therefore, it is important to impose strict monitoring and surveillance of new invasion of *B*. *straminea* in areas at high risk.

## Supporting information

S1 Table List of studies used to compile the occurrence database for *B*. *straminea*. (DOC)

S2 Table Bioclimatic variables used in the ecological niche modeling. (DOCX)

S1 Fig Pearson’s correlation matrix of all variables. (TIF)
