# Supplementary material for: Prediction of the potential global distribution for Biomphalaria straminea, an intermediate host for Schistosoma mansoni
Source: PLoS Negl Trop Dis. 2018 May 29;12(5):e0006548. doi: 10.1371/journal.pntd.0006548 (PMC5993297; doi:10.1371/journal.pntd.0006548)
Supplement: S1 Table — (DOC) [file pntd.0006548.s001.doc]

**S1 Table. List of studies used to compile the occurrence database for *B. straminea*.**

| Year | Locality | Country | Invaded or native | Study |
| --- | --- | --- | --- | --- |
| 1970 | Paraguay, Argentina | Paraguay, Argentina | Invaded | Ref 11 |
| 1987 | Corrientes Province | Argentina | Invaded | Ref 12 |
| 1987 | , Espinillar, near Salto, in the area of the Salto Grande reservoir (31°23’S, 57°58’W) | Uruguay | Invaded | Ref 13 |
| 1966 | Columbia | Columbia | Invaded | Ref 14 |
| 1976 | Coris river, Cartago Province | Costa Rica | Invaded | Ref15 |
| Around 1950 | Martinique, [Lesser Antilles](https://en.wikipedia.org/wiki/Lesser_Antilles) in the eastern [Caribbean Sea](https://en.wikipedia.org/wiki/Caribbean_Sea) | France | Invaded | Ref 16 |
| 1970 | Grenada | Grenada | Invaded | Ref 17 |
| 1985 | Guadeloupe | Guadeloupe | Invaded | Ref 18,19 |
| 1992 | St Lucia | St Lucia | Invaded | Ref 19 |
| 2006-2012 | Itamaracá Island | Brazil | Native | Ref 20 |
| 1973 | Hong Kong | China | Invaded | Ref 21 |
| 2016-2017 | Hong Kong | China | Invaded | Ref 59 |
| 1981-82 | Hong Kong | China | Invaded | Ref 60, [1] |
| 1972 | Martinique, [Lesser Antilles](https://en.wikipedia.org/wiki/Lesser_Antilles) in the eastern [Caribbean Sea](https://en.wikipedia.org/wiki/Caribbean_Sea) | France | Invaded | [2] |
| 1988 | State of Sergipe | Brazil | Native | [3] |
| 1991 | Rio Grande do Sul and Santa Catarina States | Brazil | Native | [4] |
| 1992 | Northeast (41 °W and 11°S), south of Bahia and northeast of Minas Gerais (15°-18 °S, 40-44°W) | Brazil | Native | [5] |
| 1989 | Santa Catarina State | Brazil | Invaded | [6] |
| 1992 | Martinique, French West Indies | France | Invaded | [7] |
| 1994 | Santa Catarina, | Brazil | Native | [8] |
| 1996 | Santa Lucia Reservoir, Belo Horizonte, Minas Gerais | Brazil | Native | [9] |
| 1996 | State of S. Paulo | Brazil | Native | [10] |
| 1990 | Paracambi,  State of Rio de Janeiro | Brazil |  | [11] |
| 1994-96 | Santa Lucia Park, Belo Horizonte, Minas Gerais | Brasil | Native | [12] |
| 1997 | Uberlândia, Minas Gerais | Brasil | Invaded | [13] |
| 1999 | Cachoeira Dourada, South Goiano | Brazil | Invaded | [14] |
| 2000 | Semi-arid region of Pernambuco State | Brazil | Invaded | [15] |
| 2001 | Belém, Pará (01°27’S, 48°30’W); Monte Carmelo, Minas Gerais (18°43S, 47°29’W); Passos, Minas Gerais (20°43’S, 46°36’W); São Lourenço da Mata, Pernambuco (07°58’S, 35°02’W); Brasília, Distrito Federal (15°46’S, 47°55’W); Icém, São Paulo (20°20’S, 49°11’W) | Brazil | Native | [16] |
| 1999-2000 | Minas Gerais | Brazil | Native | [17] |
| 2002 | Jaboticatubas, State of Minas Gerais (19°31’S, 43°44’W) | Brazil | Invaded | [18] |
| 1995-2000 | Niterói, Rio de Janeiro State | Brazil | Native | [19] |
| 2003 | Northern coast of the State of São Paulo | Brazil | Native | [20] |
| 1973 | Pond near Arroyo Riachuelo, Corrientes; Laguna La Guardia, Santa Fe; Tigre, Vicente López, Buenos Aires; Río Tercero at Villa Rumipal, Córdoba | Argentina | Invaded | [21] |
| 1997 | Amazonas, Acre, Roraíma, Pará, Maranhão, Piauí, Ceará, Rio Grande  do Norte, Alagoas, Paraíba, Pernambuco, Sergipe, Bahia, Minas  Gerais, Espírito Santo, Rio de Janeiro, São Paulo, Mato, Grosso, Mato  Grosso do Sul, Goiás, Distrito Federal, Paraná and Rio Grande do Sul | Brazil | Native | [22] |
| 2007 | São Francisco River Basin, Minas Gerais | Brazil | Native | [23] |
| 2005-2009 | Chiador Piau Santana,Minas Gerais | Brazil | Native | [24] |
| 2009-2010 | Ilha das Flores | Brazil | Native |  |
| 2009-2012 | Pampulha reservoir, Belo Horizonte, State of Minas  Gerais (l19°51' S, 43°58' W) | Brazil | Native | [25] |
| NA | Municipalities with records of *B. straminea* | Brazil | Native | [26] |

The prefix "Ref" represents references from the main reference list.

**References**

1. Woodruff DS, Mulvey M, Yipp MW. Population genetics of Biomphalaria straminea in Hong Kong. J Hered. 1985;76: 355-60.

2. Guyard A, Pointier JP. Freshwater snails and vectors of schistosomiasis mansoni in Martinique (French West Indies). Ann Parasitol Hum Comp. 1979;54: 193-205.

3. Figueiredo CC. Dispersion of Biomphalaria straminea in the State of Sergipe: a comparative study within an interval of 19 years. Mem Inst Oswaldo Cruz. 1989;84: 383-7.

4. Teles HM, Pereira PA, Richinitti LM. [The distribution of Biomphalaria (Gastropoda, Planorbidae) in the states of Rio Grande do Sul and Santa Catarina, Brazil]. Rev Saude Publica. 1991;25: 350-2.

5. Carvalho OD. Intermediate hosts of Schistosoma mansoni in Brazil. Mem Inst Oswaldo Cruz. 1992;87: 307-9.

6. Ferrari AA, Hofmann PR. First register of Biomphalaria straminea Dunker, 1848, in Santa Catarina State. Rev Inst Med Trop Sao Paulo. 1992;34: 33-5.

7. Pointier JP, Guyard A. Biological control of the snail intermediate hosts of Schistosoma mansoni in Martinique, French West Indies. Trop Med Parasitol. 1992;43: 98-101.

8. Schlemper JB, Ferreira NJ, Thiago PT, Bressan C, Do AA. Geographic distribution of planorbidae in Santa Catarina, Brazil. Rev Soc Bras Med Trop. 1996;29: 411-8.

9. Souza CP, Ribeiro PR, Guimaraes CT, Jannotti-Passos LK, Souza MA. Schistosomiasis: new occurrence of Biomphalaria straminea in Belo Horizonte, Minas Gerais. Cad Saude Publica. 1996;12: 541-544.

10. Teles HM. Distribution of Biomphalaria straminae in the southern neotropical region of Brazil. Rev Saude Publica. 1996;30: 341-9.

11. Da SC, Soares MS, Barreto MG. Occurrence of Biomphalaria tenagophila and disappearance of Biomphalaria straminea in Paracambi, RJ, Brazil. Mem Inst Oswaldo Cruz. 1997;92: 37-8.

12. Guimaraes CT, Souza MA, Soares D, Souza CP. A malacological survey in city parks in Belo Horizonte, Minas Gerais, Brasil. Cad Saude Publica. 1997;13: 313-316.

13. Silveira EP, Marcal JO, Machado MI. The occurrence of Biomphalaria straminea (Pulmonata: Planorbidae) on an aquaculture farm of IBAMA in Uberlandia, MG. Rev Soc Bras Med Trop. 1997;30: 401-3.

14. Marchiori CH. First occurrence of Biomphalaria straminea in the South Goiano, Brazil. Rev Saude Publica. 1999;33: 622-3.

15. Gazin P, Barbosa CS, Bouvy M, Audry P. Occurrence of snail vectors of schistosomiasis in a dam of the semi-arid region of Pernambuco State, Brazil. Rev Soc Bras Med Trop. 2000;33: 407-8.

16. Caldeira RL, Vidigal TH, Simpson AJ, Carvalho OS. Genetic variability in Brazilian populations of Biomphalaria straminea complex detected by simple sequence repeat anchored polymerase chain reaction amplification. Mem Inst Oswaldo Cruz. 2001;96: 535-44.

17. Kloos H, de Souza C, Gazzinelli A, Soares FB, Da CT, Bethony J, et al. The distribution of Biomphalaria spp. in different habitats in relation to physical, biological, water contact and cognitive factors in a rural area in Minas Gerais, Brazil. Mem Inst Oswaldo Cruz. 2001;96: 57-66.

18. Massara CL, Carvalho OS, Caldeira RL, Jannotti-Passos LK, Schall VT. First report on the presence of Biomphalaria straminea in the municipality of Jaboticatubas, State of Minas Gerais, Brazil. Mem Inst Oswaldo Cruz. 2002;97: 27-9.

19. Medeiros AS, Cruz OJ, Fernandez MA. Schistosomiasis mansoni and distribution of freshwater mollusks in natural bodies of water in Niteroi, Rio de Janeiro State, Brazil. Cad Saude Publica. 2002;18: 1463-8.

20. Teles HM, Hiramoto RM, de Oliveira JC, de Aveiro JA. Occurrence of schistosomiasis mansoni vectors on the Northern coast of the State of Sao Paulo, Brazil. Cad Saude Publica. 2003;19: 1887-91.

21. Paraense WL. Planorbidae, lymnaeidae and physidae of Argentina (mollusca: basommatophora). Mem Inst Oswaldo Cruz. 2005;100: 491-3.

22. Caldeira RL, Jannotti-Passos LK, Carvalho OS. Molecular epidemiology of Brazilian Biomphalaria: A review of the identification of species and the detection of infected snails. Acta Trop. 2009;111: 1-6.

23. Guimarães RJPS, Freitas CC, Dutra LV, Felgueiras CA, Moura ACM, Amaral RS, et al. Spatial distribution of Biomphalaria mollusks at São Francisco River Basin, Minas Gerais, Brazil, using geostatistical procedures. Acta Trop. 2009;109: 181-186.

24. Tibirica SH, Mitterofhe A, Castro MF, Lima AC, Goncalves M, Pinheiro IO, et al. Malacological survey of Biomphalaria snails in municipalities along the Estrada Real in the southeast of the State of Minas Gerais, Brazil. Rev Soc Bras Med Trop. 2011;44: 163-7.

25. Pinto HA, Mati VLT, Melo ALD. The Pampulha reservoir remains a potential urban focus of schistosomiasis mansoni in Brazil: changes in the occurrence patterns of Biomphalaria species and a new record of the parasite. Rev Soc Bras Med Tro. 2013;46: 478-483.

26. Carvalho OS, Amaral RS, Dutra LV, Scholte RGC, Guerra M. Distribuição espacial de Biomphalaria glabrata, B. straminea e B. tenagophila moluscos hospedeiros intermediários do Schistosoma mansoni no Brasil. In: Carvalho OS, Coelho PMZ, Lenzi HL, ^editors. Schistosoma mansoni e esquistossomose: uma visão multidisciplinar. Rio de Janeiro: Fiocruz; 2008. p. 395-418.
