# Supplementary material for: Prediction of the potential global distribution for Biomphalaria straminea, an intermediate host for Schistosoma mansoni
Source: PLoS Negl Trop Dis. 2018 May 29;12(5):e0006548. doi: 10.1371/journal.pntd.0006548 (PMC5993297; doi:10.1371/journal.pntd.0006548)
Supplement: S2 Table — (DOCX) [file pntd.0006548.s002.docx]

**Table S2. Bioclimatic variables used in the ecological niche modeling.**

| **Variables** | **Description** | **Sources** |
| --- | --- | --- |
| bio1 | Annual mean temperature | WorldClim database (http://www.worldclim.org/) |
| bio2 | Mean diurnal temperature range |  |
| bio3 | Isothermality |  |
| bio4 | Temperature seasonality |  |
| bio5 | Max temperature of warmest month |  |
| bio8 | Mean temperature of wettest quarter |  |
| bio12 | Annual precipitation |  |
| bio13 | Precipitation of wettest period |  |
| bio14 | Precipitation of driest period |  |
| bio15 | Precipitation seasonality |  |
| bio18 | Precipitation of warmest quarter |  |
| bio19 | Precipitation of coldest quarter |  |
| Human footprint | Anthropogenic impacts on the environment | Wildlife Conservation Society (WCS), and Center for International Earth Science Information Network (CIESIN)(http://sedac.ciesin.columbia.edu/data/collection/wildareas-v2) |
