# Supplementary figures and images for: Prediction of the potential global distribution for Biomphalaria straminea, an intermediate host for Schistosoma mansoni
Source: PLoS Negl Trop Dis. 2018 May 29;12(5):e0006548. doi: 10.1371/journal.pntd.0006548 (PMC5993297; doi:10.1371/journal.pntd.0006548)

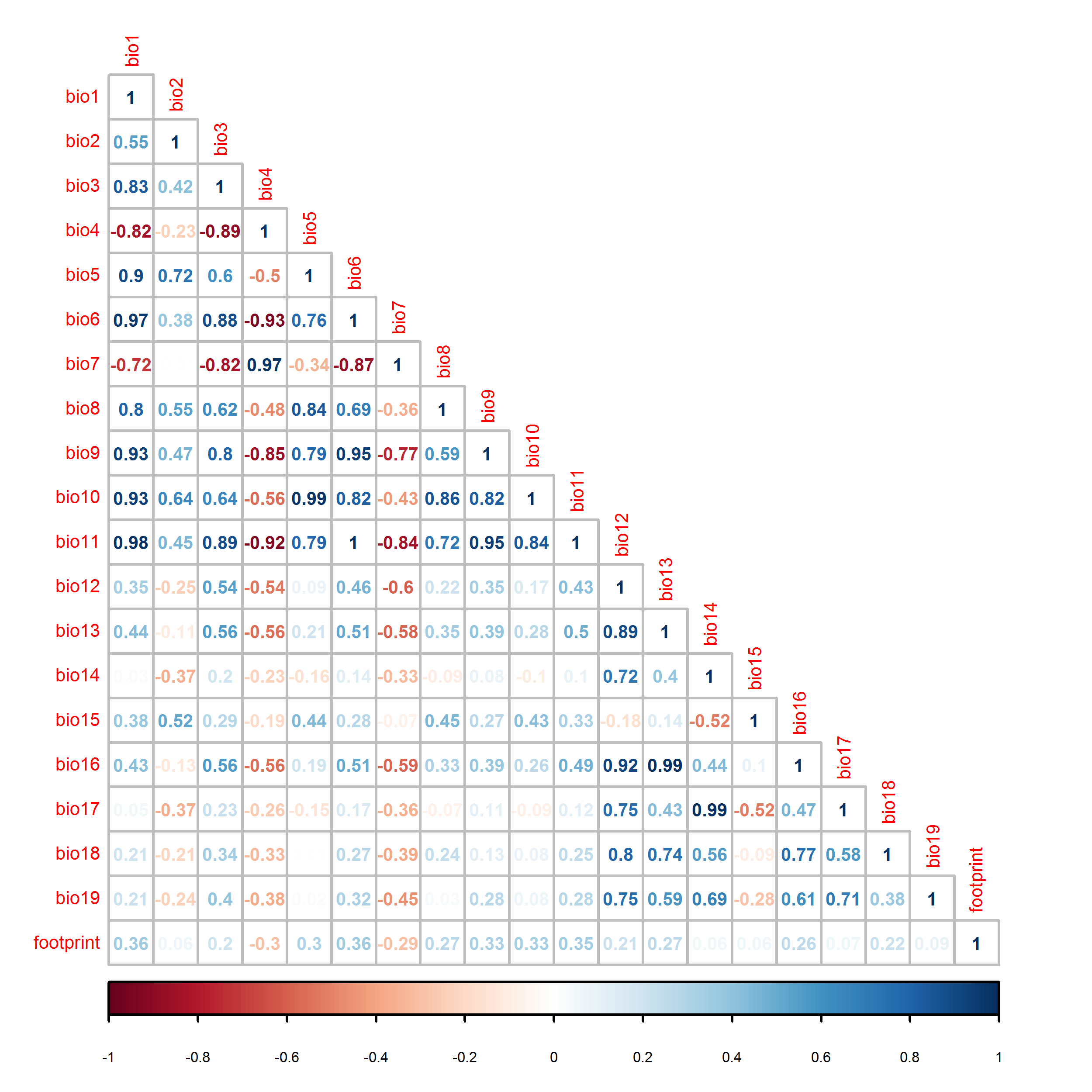

Supplement: S1 Fig — (TIF) [file pntd.0006548.s003.tif]
